# Supplementary material for: Resolving the full spectrum of human genome variation using Linked-Reads
Source: Genome Res. 2019 Apr;29(4):635–45. doi: 10.1101/gr.234443.118 (PMC6442396; doi:10.1101/gr.234443.118)
Supplement: Supplemental Material [file supp_gr.234443.118_Supplemental_longranger-2.2.zip › longranger-2.2.tar.bz2!/lr2.2-public/lib/stan/cmdstan/stan/doc/api/html/model_2util_8hpp.html]

Stan: src/stan/model/util.hpp File Reference


|  |  |
| --- | --- |
|  | Stan  2.5.0  probability, sampling & optimization |


- Main Page
- Related Pages
- Namespaces
- Classes
- Files

- File List
- File Members

All Classes Namespaces Files Functions Variables Typedefs Enumerations Enumerator Friends Macros Pages

- src
- stan
- model

Classes |
Namespaces |
Functions

util.hpp File Reference

`#include <cmath>`  
`#include <iomanip>`  
`#include <iostream>`  
`#include <vector>`  
`#include <stan/math/matrix/Eigen.hpp>`  
`#include <stan/agrad/rev/matrix/grad.hpp>`  
`#include <stan/agrad/rev/var.hpp>`  
`#include <stan/agrad/autodiff.hpp>`

Go to the source code of this file.

|  |  |
| --- | --- |
| Classes | |
| struct | stan::model::model\_functional< M > |
|  | |

|  |  |
| --- | --- |
| Namespaces | |
|  | stan |
|  | Probability, optimization and sampling library. |
|  | |
|  | stan::model |
|  | For compiling models. |
|  | |

|  |  |
| --- | --- |
| Functions | |
| template<bool jacobian\_adjust\_transform, class M > | |
| double | stan::model::log\_prob\_propto (const M &model, std::vector< double > &params\_r, std::vector< int > &params\_i, std::ostream \*msgs=0) |
|  | Helper function to calculate log probability for `double` scalars up to a proportion. More... |
|  | |
| template<bool propto, bool jacobian\_adjust\_transform, class M > | |
| double | stan::model::log\_prob\_grad (const M &model, std::vector< double > &params\_r, std::vector< int > &params\_i, std::vector< double > &gradient, std::ostream \*msgs=0) |
|  | Compute the gradient using reverse-mode automatic differentiation, writing the result into the specified gradient, using the specified perturbation. More... |
|  | |
| template<bool jacobian\_adjust\_transform, class M > | |
| double | stan::model::log\_prob\_propto (const M &model, Eigen::VectorXd &params\_r, std::ostream \*msgs=0) |
|  | Helper function to calculate log probability for `double` scalars up to a proportion. More... |
|  | |
| template<bool propto, bool jacobian\_adjust\_transform, class M > | |
| double | stan::model::log\_prob\_grad (const M &model, Eigen::VectorXd &params\_r, Eigen::VectorXd &gradient, std::ostream \*msgs=0) |
|  | Compute the gradient using reverse-mode automatic differentiation, writing the result into the specified gradient, using the specified perturbation. More... |
|  | |
| template<bool propto, bool jacobian\_adjust\_transform, class M > | |
| void | stan::model::finite\_diff\_grad (const M &model, std::vector< double > &params\_r, std::vector< int > &params\_i, std::vector< double > &grad, double epsilon=1e-6, std::ostream \*msgs=0) |
|  | Compute the gradient using finite differences for the specified parameters, writing the result into the specified gradient, using the specified perturbation. More... |
|  | |
| template<bool propto, bool jacobian\_adjust\_transform, class M > | |
| int | stan::model::test\_gradients (const M &model, std::vector< double > &params\_r, std::vector< int > &params\_i, double epsilon=1e-6, double error=1e-6, std::ostream &o=std::cout, std::ostream \*msgs=0) |
|  | Test the log\_prob\_grad() function's ability to produce accurate gradients using finite differences. More... |
|  | |
| template<bool propto, bool jacobian\_adjust\_transform, class M > | |
| double | stan::model::grad\_hess\_log\_prob (const M &model, std::vector< double > &params\_r, std::vector< int > &params\_i, std::vector< double > &gradient, std::vector< double > &hessian, std::ostream \*msgs=0) |
|  | Evaluate the log-probability, its gradient, and its Hessian at params\_r. More... |
|  | |
| template<class M > | |
| void | stan::model::gradient (const M &model, const Eigen::Matrix< double, Eigen::Dynamic, 1 > &x, double &f, Eigen::Matrix< double, Eigen::Dynamic, 1 > &grad\_f, std::ostream \*msgs=0) |
|  | |
| template<class M > | |
| void | stan::model::hessian (const M &model, const Eigen::Matrix< double, Eigen::Dynamic, 1 > &x, double &f, Eigen::Matrix< double, Eigen::Dynamic, 1 > &grad\_f, Eigen::Matrix< double, Eigen::Dynamic, Eigen::Dynamic > &hess\_f, std::ostream \*msgs=0) |
|  | |
| template<class M > | |
| void | stan::model::gradient\_dot\_vector (const M &model, const Eigen::Matrix< double, Eigen::Dynamic, 1 > &x, const Eigen::Matrix< double, Eigen::Dynamic, 1 > &v, double &f, double &grad\_f\_dot\_v, std::ostream \*msgs=0) |
|  | |
| template<class M > | |
| void | stan::model::hessian\_times\_vector (const M &model, const Eigen::Matrix< double, Eigen::Dynamic, 1 > &x, const Eigen::Matrix< double, Eigen::Dynamic, 1 > &v, double &f, Eigen::Matrix< double, Eigen::Dynamic, 1 > &hess\_f\_dot\_v, std::ostream \*msgs=0) |
|  | |
| template<class M > | |
| void | stan::model::grad\_tr\_mat\_times\_hessian (const M &model, const Eigen::Matrix< double, Eigen::Dynamic, 1 > &x, const Eigen::Matrix< double, Eigen::Dynamic, Eigen::Dynamic > &X, Eigen::Matrix< double, Eigen::Dynamic, 1 > &grad\_tr\_X\_hess\_f, std::ostream \*msgs=0) |
|  | |


---

[ Stan Home Page ]

*© 2011–2014,
Stan Development Team.*
